# Methylation of the D2 dopamine receptor affects binding with the human regulatory proteins Par-4 and Calmodulin

**DOI:** 10.17912/micropub.biology.000366

**Published:** 2021-02-09

**Authors:** Alexander Bowitch, Ansuman Sahoo, Andrea M. Clark, Christiana Ntangka, Krishna K. Raut, Paul Gollnick, Michael C. Yu, Steven M. Pascal, Sarah E. Walker, Denise M. Ferkey

**Affiliations:** 1 Department of Biological Sciences, University at Buffalo, The State University of New York, Buffalo, NY 14260; 2 Department of Chemistry and Biochemistry, Old Dominion University, Norfolk, VA 23529; 3 Current address: Department of Biochemistry and Biophysics, Brandeis University, Waltham, MA 02454

**Figure 1. Binding of unmethylated and methylated D2 peptide to Prostate Apoptosis Response 4 Protein (Par-4) and Calmodulin (CaM) in anisotropy experiments:**
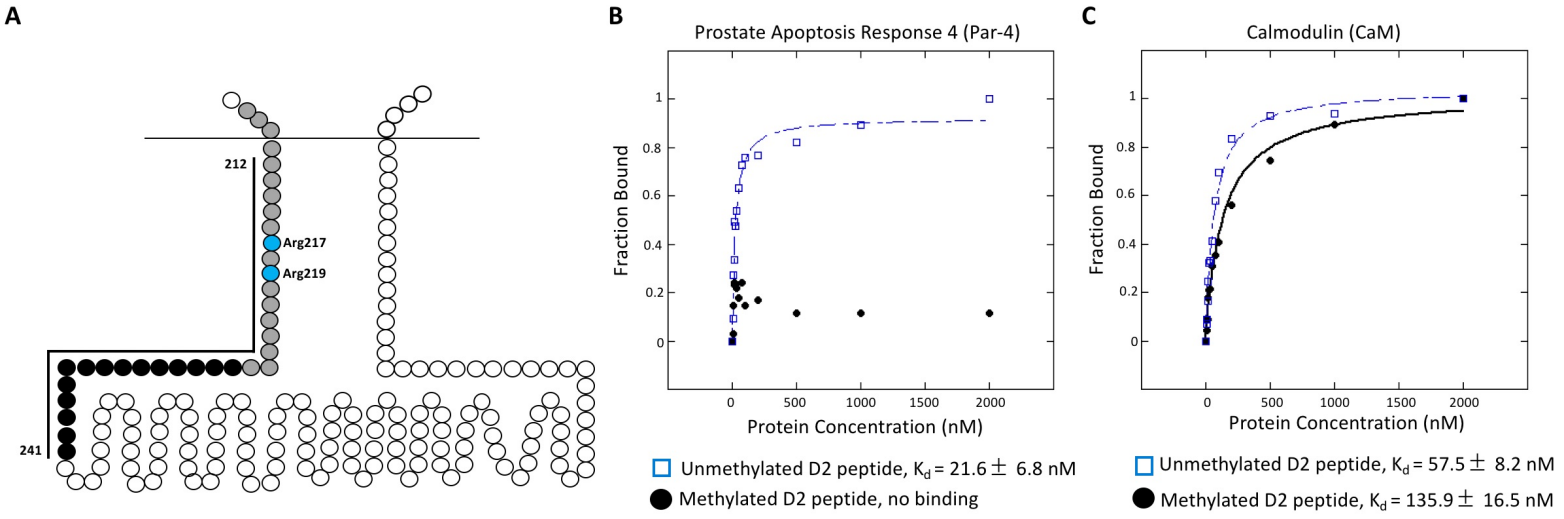
(A) Human D2 receptor third intracellular loop (3^rd^ ICL). Methylated arginines are indicated in blue (Arg 217 and Arg 219). Grey circles represent the CaM binding site (residues 208-226), while grey and black circles (indicated by the line) represent the Par-4 binding site (residues 212-241). The D2 peptide used for anisotropy corresponded to amino acids 208-243. (B) Fraction of human Par-4 protein bound to either unmethylated or methylated D2 peptide. The graph represents the pooled data of four individual experiments. (C) Fraction of human CaM protein bound to either unmethylated or methylated D2 peptide. The graph represents the pooled data of four individual experiments. The K_d_ values shown in each panel are an average of four separate anisotropy trials, plus or minus the standard error of the mean. KaleidaGraph was used to plot the best fit curves and to determine the binding constants (K_d_).

## Description

Protein arginine methylation is a post-translational modification that contributes to the regulation of many cellular processes, including DNA damage repair, transcriptional regulation and protein-protein interaction (Bedford and Richard 2005). In 2003, a large-scale proteomics analysis identified a number of G protein-coupled receptors (GPCRs) as potentially being arginine methylated (Boisvert *et al.* 2003). However, the effect of methylation on these receptors was not examined. Functional evidence that protein arginine methylation regulates GPCR signaling was first reported for the D2-like dopamine GPCR family (the human D2 and *C. elegans* DOP-3 receptors) (Likhite *et al.* 2015), and later for the *C. elegans* SER-2 tyramine receptor (Bowitch *et al.* 2018). Protein arginine methyltransferase 5 (PRMT5) methylated a peptide fragment corresponding to a region of the third intracellular loop (3^rd ^ICL) of each of these receptors *in vitro*, while changing the conserved arginines in this region to alanine reduced receptor methylation (Likhite *et al.*2015; Bowitch *et al.* 2018). In cell culture, mutating the conserved putative methylation motif of human D2 decreased signaling via the receptor, leading to a decrease in D2-mediated inhibition of cAMP (Likhite *et al.* 2015). This, combined with behavioral studies in *C. elegans*, suggested that protein arginine methylation promotes D2-like dopamine receptor signaling (Likhite *et al.* 2015). However, the mechanism by which it does so is unknown.

The two arginine residues (Arg217 and Arg219) that we identified as methylation targets within the amino-terminal region of the 3^rd ^ICL of human D2 (Likhite *et al.* 2015) lie within the overlapping binding sites of Par-4 [prostate apoptosis response 4 protein, also known as pro-apoptotic WT1 regulator (PAWR)] and CaM (calmodulin) (Bofill-Cardona *et al.* 2000; Park *et al.* 2005) (Figure 1A). The two proteins compete for binding to D2, with CaM displacing Par-4 in a calcium-dependent manner (Park *et al.* 2005). Whereas Par-4 binding facilitated D2 inhibition of adenylyl cyclase (Park *et al.* 2005), CaM binding to D2N (a peptide corresponding to the first 19 amino acids of D2’s third intracellular loop) selectively blocked G protein activation (Bofill-Cardona *et al.* 2000). The opposing activities for Par-4 and CaM are consistent with their competition for binding to D2.

We sought to determine whether arginine methylation could regulate D2 signaling by altering the binding of Par-4 and/or CaM to D2. Specifically, as arginine methylation of D2 was proposed to promote D2 signaling (Likhite *et al.*2015), we hypothesized that it might either enhance Par-4 binding, or diminish CaM binding, to D2. We performed anisotropy experiments using purified recombinant human Par-4 or human CaM, with fluorescently-labeled unmethylated or methylated D2 peptide (corresponding to amino acids 208-243 of human D2), to determine binding constants. Unexpectedly, although Par-4 bound to the unmethylated D2 peptide with an average K_d_ = 21.6 ± 6.8 nM, no binding was observed in the presence of the methylated D2 peptide (Figure 1B). This suggests that methylation of the D2 receptor may actually serve to abrogate its interaction with Par-4, perhaps to allow binding of an alternative (unidentified) promoter of D2 signaling. However, consistent with the model that arginine methylation may modulate D2 receptor signaling in part by decreasing its interaction with CaM, we found that CaM bound more strongly to the unmethylated D2 peptide (average K_d_ = 57.5 ± 8.2 nM) than to the methylated D2 peptide (average K_d_ = 135.9 ± 16.5 nM).

Combined, we have demonstrated that the methylation status of a peptide fragment of the 3^rd ^ICL of D2 influences interaction with two known modulators of D2 receptor signaling, Par-4 and CaM, *in vitro*. Future cell-based experiments to examine the effect of arginine methylation on full-length D2 receptor interaction with cytoplasmic regulators will provide additional insights into the mechanism by which this post-translational modification may promote D2-mediated dopamine signaling.

## Methods

Peptide synthesis

Amino-terminal FLAG-tagged peptide corresponding to amino acids 208-243 of the third intracellular loop of the human D2 receptor was synthesized by 21^st^ Century Biochemicals with a K-5-FAM (Carboxyfluorescein) fluorophore at the carboxy-terminal end. Methylated peptides were symmetrically dimethylated at the arginines corresponding to Arg 217 and Arg 219 of D2.

His-Par-4 purification

Amino-terminal His-tagged human Par-4 was purified as previously described (Clark *et al.* 2018) and dialyzed into 200 mM NaCl, 200 µM CaCl_2_, 50 mM Tris pH 7.5 prior to lyophilization. The protein sample was reconstituted with water for use in anisotropy experiments.

His-Calmodulin purification

Amino-terminal His-tagged human calmodulin purification was adapted from (McCluskey *et al.* 2007). Rosetta *E. coli* transformants harboring the His-Calmodulin (in pET-17b, Novagen) plasmid were grown in Luria broth (LB) containing 100 µg/mL ampicillin. Cells were grown in shaking flasks at 37°C until they reached an OD_600_ of 0.6. Protein expression was induced with 0.75 mM IPTG and cultures were maintained overnight with shaking at room temperature. Cells were pelleted at 6000 x g for 15 minutes, washed once with PBS, and then stored at -80°C. Cell pellets from a 1-liter culture were resuspended in 30 mL of Buffer A (50 mM Tris-HCl pH 7.5, 1 mM CaCl_2_, 1 mM PMSF). Cells were lysed by two rounds of French press, cleared by centrifugation at 30,000 x g for 30 minutes and then filtered. Sample was adjusted to 10 mM imidazole and then loaded onto a 2-mL bed of Ni-NTA beads (Qiagen) that had been pre-equilibrated with 10 mL of Buffer A. Beads were washed three times with 10 mL of Buffer A. Sample was then eluted with 10 mL of Buffer A that had been adjusted to 150 mM imidazole. The elution was directly applied to a 2-mL bed of Phenyl-sepharose beads (Qiagen) that had been pre-equilibrated with Buffer A. Beads were washed three times with 10 mL of buffer A, and the sample was eluted in 5 mL of buffer B (50 mM Tris-HCl pH 7.5, 5 mM EGTA), and titrated with 50 mL of 1 M CaCl_2_. Protein was dialyzed into 1x TBS/15% glycerol overnight and stored at -80°C.

Anisotropy

10x stock concentrations of CaM or Par-4 were made in binding buffer (200 mM NaCl, 200 µM CaCl_2_, 50 mM Tris pH 7.5) at concentrations ranging from 0-20 µM. A master mix was made with binding buffer and fluorescently labeled unmethylated or methylated D2 peptide to yield a final concentration of 0.025 µM. 22.5 µL of master mix was aliquoted to each tube, and then 2.5 µL of the 10x stock concentration of either Par-4 or Calmodulin was added. Solutions were mixed by pipetting up and down three times and allowed to incubate at room temperature for ten minutes. 10 µL of each sample were pipetted into two adjacent wells in a 384-well black plate (Corning 3820), and fluorescence polarization was read in a SpectraMax i3x plate reader. K_d_ values were determined using KaleidaGraph.

## References

[R1] Bedford MT, Richard S (2005). Arginine methylation an emerging regulator of protein function.. Mol Cell.

[R2] Bofill-Cardona E, Kudlacek O, Yang Q, Ahorn H, Freissmuth M, Nanoff C (2000). Binding of calmodulin to the D2-dopamine receptor reduces receptor signaling by arresting the G protein activation switch.. J Biol Chem.

[R3] Boisvert FM, Côté J, Boulanger MC, Richard S (2003). A proteomic analysis of arginine-methylated protein complexes.. Mol Cell Proteomics.

[R4] Bowitch A, Michaels KL, Yu MC, Ferkey DM (2018). The Protein Arginine Methyltransferase PRMT-5 Regulates SER-2 Tyramine Receptor-Mediated Behaviors in *Caenorhabditis elegans*.. G3 (Bethesda).

[R5] Clark AM, Ponniah K, Warden MS, Raitt EM, Yawn AC, Pascal SM (2018). pH-Induced Folding of the Caspase-Cleaved Par-4 Tumor Suppressor: Evidence of Structure Outside of the Coiled Coil Domain.. Biomolecules.

[R6] Likhite N, Jackson CA, Liang MS, Krzyzanowski MC, Lei P, Wood JF, Birkaya B, Michaels KL, Andreadis ST, Clark SD, Yu MC, Ferkey DM (2015). The protein arginine methyltransferase PRMT5 promotes D2-like dopamine receptor signaling.. Sci Signal.

[R7] McCluskey AJ, Poon GM, Gariépy J (2007). A rapid and universal tandem-purification strategy for recombinant proteins.. Protein Sci.

[R8] Park SK, Nguyen MD, Fischer A, Luke MP, Affar el B, Dieffenbach PB, Tseng HC, Shi Y, Tsai LH (2005). Par-4 links dopamine signaling and depression.. Cell.

